# Resilience and Suicidal Thoughts in Young People: Based on the Rafsanjan Youth Cohort Study

**DOI:** 10.34172/aim.2023.92

**Published:** 2023-11-01

**Authors:** Marjan Sadeghi, Hassan Ahmadinia, Fatemeh Ayoobi, Mohsen Rezaeian

**Affiliations:** ^1^Non-Communicable Diseases Research Center, Rafsanjan University of Medical Sciences, Rafsanjan, Iran; ^2^Department of Epidemiology and Biostatistics, School of Health, Occupational Environment Research Center, Rafsanjan University of Medical Sciences, Rafsanjan, Iran

**Keywords:** Prospective epidemiological research studies in IRAN (PERSIAN), Rafsanjan cohort study (RCS), Resilience, Suicide, Youth

## Abstract

**Background:**

Suicide is a serious public health problem, and suicide attempt is defined as one of the important indicators of mental health in a society. The present study investigated the relationship between resilience and suicidal thoughts in Rafsanjani youth.

**Methods:**

This cross-sectional descriptive study examined 3006 young people aged 15-35 who referred to Rafsanjan Cohort Study (RCS). Connor and Davidson’s questionnaire was used to measure resilience. The data, including demographic characteristics and cases related to suicide, were extracted from the computer system, which is part of the Rafsanjan Youth Cohort Program. Independent *t*-test, one-way analysis of variance, Tukey’s post-hoc test, Pearson’s correlation coefficient and multiple linear regression model were used to analyze the data.

**Results:**

A total of 3006 individuals were studied, of whom 1685 (56.1%) were women, and 1321 (43.9%) were men. The average age of the participants was 25.75±6.09 years. Men had a significantly higher average score of resilience (*P*<0.001) and suicidal thoughts (*P*=0.002) than women. In addition, the average score of suicidal thoughts in divorced and widowed people was significantly higher than single and married people (*P*=0.029). It was shown that older age groups had higher average resilience (*P*<0.001) and fewer suicidal thoughts (*P*=0.003), and people over 30 years had the lowest average suicidal thoughts.

**Conclusion:**

The results indicate an inverse and significant relationship between resilience and suicidal thoughts in both men and women. Therefore, more evaluations are necessary to investigate the factors affecting resilience and take measures to improve it among young people.

## Introduction

 Suicide is a serious public health problem, and suicide attempt is one of the important indicators of mental health in a society. The term suicide is defined as harming oneself to destroy oneself. There are many reasons for death by suicide.^1^ Suicide is a phenomenon that can be defined by a set of balanced and entwined factors, and none of its single-causal explanations can correctly explain its multifaceted nature.^2^ Suicide occurs among all age groups, but the important point is that suicide causes the loss of more potential years in the younger generation. When young people are dealing with problems that they are not familiar with, their mental health is negatively affected. It is also very concerning for the family and society from a psychological point of view.^3,4^ When young people do not know how to deal with their problems, they may be drawn to suicide.^5^ Despite the lack of global epidemiologic data for adolescents, suicide is still one of the main causes of death worldwide. It is also one of the major concerns in public health.^6,7^ Southeast Asia and Eastern Europe have the highest rates of adolescent suicide in both sexes.^8^ Approximately 4600 young people between the ages of 10 and 24 yearly lost their lives by suicide. Shockingly, there are between 100 and 200 suicide attempts for every suicide that leads to death in teenagers aged 15 to 34.^9^ Research on suicide in Iran indicates that the age range was 30-39 for men, under 30 for women. Suicidal thoughts and attempts are closely related to people’s personalities and mental states.

 Resilience is a new concept in this context that has recently received attention. One of the research interests in the resilience field is determining the resilience factors according to individual, family and social characteristics. Resilience is a psychological concept that describes how people cope with unexpected situations. Resilience means persistence against stress, the ability to return to a normal situation, survive, and strive during confrontational conditions.^10^ According to Davidson and Connor, resilience is a person’s ability to maintain biological and psychological balance in dangerous situations. They believe that resilience is not persisting in threatening situations; rather, the person’s active participation in the environment is more important.^11^ All definitions of resilience have several aspects in common despite their different definitions. Resilience is first defined as a level of coping with difficulties. In other words, resilience is people’s responsiveness to problems. Second, resilience brings a feeling of lightness and comfort. On the other hand, individuals present resilience as the ability to repair adversity and influence and overcome adversity based on their different levels of functioning. Ultimately, resilience is a superior ability that allows people to overcome difficulty and hardship. Nowadays, resilience has a superior place in the fields of mental health and evolutionary psychology. In addition, it has been a significant construct in well-being theories and research for more than two decades.^12^ Therefore, the present study was conducted to determine the relationship between resilience and suicidal thoughts in young people.

## Materials and Methods

 The population in this cross-sectional study included all people aged 15‒35 years participating in the Rafsanjan Cohort Study (RCS), which is equal to 3006 people. This study is part of the prospective epidemiological research studies in IrAN (PERSIAN) whose profile is available in the American Journal of Epidemiology.^13^ It must be noted that the participants in the Persian cohort study were randomly selected using the health records of the urban and rural population of Rafsanjan. Comprehensive questionnaires were administered by trained interviewers in the phase of baseline data collection from December 2016 to December 2018. Follow-up visits are currently in progress.

 The inclusion criteria for the study included agreement to participate, absence of severe physical and mental disorders interfering with the interview process, and living in Rafsanjan city. The exclusion criteria were unwillingness to continue cooperation. The researchers received the desired data that was digitally collected after obtaining ethical approval (IR.RUMS.REC.2020.046) from Rafsanjan University of Medical Sciences. The data was not used for other purposes except for what is mentioned in this study. Demographic information included age, sex, level of education, marital status, and occupation.

 The data collection tool consisted of two comprehensive questionnaires used in the study of the Rafsanjan youth cohort. The interviews were conducted face-to-face by trained interviewers, and the individual’s answers were entered directly into the computer. The other information collected in the adult cohort study was related to the participants’ age, gender, level of education, marital status and occupation.

 Connor and Davidson’s resilience questionnaire was used to assess resilience. This questionnaire has 25 items. The scoring of this scale is based on a Likert scale between zero (completely false) and four (always true). The total scores of all questions were added together to obtain the overall score of the questionnaire. Then, the final score was calculated and analyzed in the range of 0 to 100. Higher scores indicate greater resilience. The cut-off point of this questionnaire is 50. In other words, a score higher than 50 indicates people with resilience. The higher this score is above 50, the higher the person’s resilience and vice versa. Mohammadi has standardized this scale in Iran.^14^ In a study by Abdi et al, the reliability of the questionnaire was reported at 0.83 using Cronbach’s alpha coefficient test and 0.93 using the test-retest method. The questionnaire’s construct validity has been also investigated and confirmed using confirmatory factor analysis.^15^ A questionnaire was used to evaluate suicidal thoughts and attempts in Golestan and Fasa cohorts, and its validity and reliability were confirmed.^16^ The suicide questionnaire has 9 questions whose validity and reliability have been confirmed in the Golestan cohort. The score of suicidal thoughts is also obtained from the total scores of these 9 questions and then placed in the range of 0 to 100. The Kolmogorov-Smirnov test and skewness and kurtosis indices were used to investigate the assumption of normality of these variables after calculating the scores of resilience and suicidal thoughts for each person. The normality assumption of resilience and suicidal thoughts variables was confirmed according to the non-significance of this test and the skewness and kurtosis coefficients being in the range of -1 to 1. The data were finally reported as number (percentage) and mean (standard deviation). In addition, independent t-test, one-way analysis of variance, Tukey’s post hoc test, Pearson’s correlation coefficient and multiple linear regression model were used in SPSS version 20 to analyze the data. The significance level in all tests was considered at 0.05.

## Results

 A total of 3006 individuals were studied, of whom 1683 (56%) were women, and 1323 (44%) were men. The average age of the participants was 25.75 ± 6.09 years. In this population, 1630 people (54.2%) were married, and 1212 people (40.3%) were employees. Table 1 reports the frequency distribution of different variables of the participants.

**Table 1:** Frequency Distribution of Demographic Variables in Individuals Participating in the Study

**Variable**	**Variable Level**	**Frequency**	**Percent**
Gender	Female	1683	56.0
	Male	1323	44.0
Marital status	Single	1327	44.2
	married	1630	54.2
	Other (divorced/widow)	49	1.6
Job	Unemployed	244	8.1
	Housekeeper	803	26.7
	University student	715	23.8
	Employee	1212	40.3
	Soldier	32	1.1
Education level	Elementary	62	2.1
	Middle School degree	221	7.3
	Diploma	1584	52.7
	University	1139	37.9
Age groups	15 to 20 years	750	25.0
	21 to 25 years	586	19.5
	26 to 30 years	701	23.3
	Over 30 years	898	29.9
	Unknown	71	2.3
Number of suicide attempts	None	2862	95.2
	1 to 3 times	130	4.3
	More than three times	14	.5

Table 2 compares the average resilience score at different demographic variable levels. According to the independent *t*-test, the average resilience score in men was significantly higher than women (*P* < 0.001). According to the one-way analysis of variance, no significant difference was observed across the three groups of single, married and others people (including divorced and widowed) in terms of the mean resilience score (*P* = 0.103). However, the resilience score had a significant relationship with occupation (*P* < 0.001), education level (*P* = 0.044), age groups (*P* < 0.001) and number of suicide attempts *(P* < 0.001).

**Table 2:** Comparison of Participants’ Average Resilience Score According to Different Demographic Variables

**Variable**	**Variable Level**	**Frequency**	**Mean**	**SD**	***P *** **Value**
Gender	Female	1683	59.92	20.28	< 0.001^*^
	Male	1323	65.80	19.37	
Marital Status	Single	1327	63.10	19.68	0.103^**^
	married	1630	62.17	20.33	
	Other (divorced/widow)	49	57.60	22.88	
Job	Unemployed	244	58.39	20.33	< 0.001^**^
	Housekeeper	803	59.38	21.10	
	University student	715	62.38	19.47	
	Employee	1212	65.34	19.29	
	Soldier	32	67.42	20.07	
Education Level	Elementary	62	62.14	23.07	0.044^**^
	Middle School degree	221	63.71	22.04	
	Diploma	1584	61.54	20.47	
	University	1139	63.63	18.93	
Age Groups	15 to 20 years	750	59.91	19.56	< 0.001^**^
	21 to 25 years	586	61.06	19.28	
	26 to 30 years	701	63.43	20.76	
	Over 30 years	898	64.71	20.26	
Number of suicide attempts	None	2862	63.14	19.81	< 0.001^**^
	1 to 3 times	130	50.92	22.20	
	More than three times	14	41.25	15.21	

* Independent *t* test; ** One-way analysis of variance (ANOVA)

 Also, according to Tukey’s post hoc test, the average resilience in unemployed people was significantly lower than students (*P* = 0.045) and employees (*P*< 0.001). Also, the average resilience in the housewives group was lower than students (*P* = 0.028) and employees (*P* < 0.001), and the average resilience in the students group was lower than employed people (*P* = 0.014). The average resilience in people with a diploma degree was significantly lower than university educated people (*P* = 0.036). The average resilience in people aged 15 to 20 years was significantly lower than that of people in the age group of 26 to 30 years (*P* = 0.005) and over 30 years of age (*P*< 0.001). Also, the average score of resilience in the age group of 21 to 25 years was significantly lower than those over 30 years of age (*P* = 0.003). The average resilience in people with no history of suicide was significantly higher than those with a history of 1 to 3 suicide attempts (*P* < 0.001) and those with more than 3 suicide attempts (*P* < 0.001). In other words, people with less resilience were more likely to carry out suicide. A total of 2131 participants (70.9%) had acceptable resilience (score above 50), and the remaining 875 people (29.1%) had moderate to low resilience.

Table 3 compares the average scores of suicidal thoughts at different levels of demographic variables. According to the independent t-test, the average score of suicidal thoughts in women was significantly higher than men (*P*= 0.002). According to the one-way analysis of variance, the score of suicidal thoughts had a significant relationship with marital status (*P* = 0.029), education level (*P* = 0.037), age groups (*P* = 0.003) and number of suicide attempts (*P* < 0.001). Also, according to Tukey’s post hoc test, the average score of suicidal thoughts in the other group (including divorced and widowed) was significantly higher than both single (*P* = 0.022) and married (*P* = 0.023) groups. The average score of suicidal thoughts in people with a diploma degree was significantly higher than university educated people (*P* = 0.019). The average score of suicidal thoughts in people aged 15 to 20 years was significantly higher than people over 30 years of age (*P* = 0.001). In addition, the average score of suicidal thoughts in people with more than 3 times suicide attempts was higher than those with a history of 1 to 3 suicide attempts (*P* < 0.001) and more than people with no suicide attempts (*P*< 0.001). This shows that people with more suicidal thoughts were more likely to attempt suicide.

**Table 3:** Comparison of Participants’ Average Score of Suicidal Thoughts According to Different Demographic Variables

**Variable**	**Variable Level**	**Frequency**	**Mean**	**SD**	***P *** **Value**
Gender	Female	1683	11.56	18.81	0.002^*^
	Male	1323	9.52	17.21	
Marital status	Single	1327	10.52	17.86	0.029^**^
	married	1630	10.58	18.12	
	Other (divorced/widow)	49	17.49	24.96	
Job	Unemployed	244	12.24	18.28	0.309^**^
	Housekeeper	803	11.10	18.83	
	University student	715	10.91	17.95	
	Employee	1212	9.98	17.72	
	Soldier	32	8.04	20.18	
Education level	Elementary	62	10.14	19.93	0.037^**^
	Middle school degree	221	10.86	19.17	
	Diploma	1584	11.52	19.00	
	University	1139	9.47	16.51	
Age groups	15 to 20 years	750	12.55	19.73	0.003^**^
	21 to 25 years	586	10.94	18.42	
	26 to 30 years	701	10.51	17.97	
	Over 30 years	898	9.22	16.81	
Number of suicide attempts	None	2861	7.55	11.30	< 0.001^**^
	1 to 3 times	130	71.21	17.88	
	More than three times	14	84.69	11.84	

*Independent *t* test; **One-way analysis of variance (ANOVA).

 Finally, Pearson’s correlation coefficient test was used to investigate the relationship between resilience and suicidal thoughts. The correlation coefficient between these two variables was -0.286, which indicates a significant inverse relationship. In other words, when the person’s resilience increases, the number of suicidal thoughts decreases significantly (*P* < 0.001). This coefficient was also calculated according to gender, as reported in Table 4.

**Table 4:** Correlation Coefficient Between Resilience Scores and Suicidal Thoughts According to Gender in Young People Participating in the Rafsanjan Cohort Study

**Gender**	**r**	***P*** ** Value**
Male	-.287	< 0.001
Female	-.232	< 0.001
Total	-.286	< 0.001

 Finally, in order to investigate the effect of resilience score on suicidal thoughts by adjusting other variables, a multiple linear regression model was used. All the variables that had a significant relationship with the suicidal thoughts score (including gender, marital status, education level, age and number of suicide attempts) were entered into the model, and using the backward method, only significant variables remained in the model. According to the results of this model, by adjusting other variables, with each unit increase in the resilience score, the score of suicidal thoughts decreased by 0.154 points (*P* < 0.001). Also, with each year increase in age, the suicidal thoughts score decreased by 0.109 points (*P* = 0.002). On average, this score was lower in men than women (*P*< 0.001), and with increasing history of suicide attempts, the suicidal thoughts score also increased on average (*P* < 0.001). Regression coefficients, standard error, and significance level are reported for each variable in Table 5. These findings indicate that people with more suicidal thoughts were more likely to carry out suicide.

**Table 5:** Examining the Simultaneous Effect of Different Variables on Suicidal Thoughts

**Variables**	B*	**Standartd Error**	***P *** **Value**
Resilience score	-.154	0.011	< 0.001
Sex	-1.494	0.445	< 0.001
Age	-.109	0.035	0.002
Number of suicide attempts	53.727	0.882	< 0.001

* Unstandardized coefficients.

## Discussion

 The present study was conducted to investigate the relationship between resilience and suicidal thoughts in the youth of Rafsanjani. A total of 3006 individuals were studied, of whom 56.1% were women and 43.9% were men. The participants’ average age was 25.75 years. This study showed that women’s resilience was significantly lower than men’s. Contrary to our study results, Habibi and his colleagues found no difference between boys and girls in terms of resilience. In other words, gender and resilience were not related to each other.^17^ In addition, age affects the relationship between these two variables. The current study population was young people aged 15‒35, while Habibi’s study was conducted on high school students.^17^ Furthermore, Henley did not report a significant difference between the girls’ and boys’ resilience levels but stated that girls and boys use different strategies for their resilience.^18^

 In the present study, suicidal thoughts, suicide plans and suicide attempts had a significant inverse correlation with resilience. Karami et al also indicated that the resilience score correlates inversely with suicidal thoughts in high school girls.^19^ Another study by Zarei (2021) showed that resilience had an inverse relationship with suicidal thoughts and could be used as the strongest predictor of suicide risk.^20^ Heidarisharaf et al also showed that life quality tests, resilience and spirituality can predict suicidal thoughts.^21^ According to the results of Shahbaziyan Khonig et al, social intelligence and resilience are significantly responsible for reducing the possibility of student suicide. Therefore, one must pay attention to social intelligence and resilience when it comes to preventing and reducing student suicide.^22^

 A study by Karami et al indicated that extroversion and resilience personality traits correlate negatively with the attitude towards suicide.^19^ Esfandiar et al reported that resilience training effectively improves soldiers’ stress symptoms, self-harm, and resilience and can be used in garrison environments.^23^ Sher believes that mental health specialists who actively focus on increasing stress resilience using psychosocial and pharmacological interventions are likely to reduce suicidal risk in psychiatric patients.^24^ Izadinia et al showed that suicidal thoughts had a significant and negative relationship with resilience. Anxiety, depression, mental health and daily stress correlated positively with suicidal thoughts. This study’s regression analysis indicated that depression was the most dominant factor in predicting suicidal thoughts. Anxiety, mental health, resilience and daily stress were the next contributory factors, respectively.^25^ Roy et al asked 100 drug-dependent patients if they had ever attempted suicide. The results indicated that the resilience scale scores of patients with suicide attempts (41 subjects) were significantly lower than those without suicide attempts (59 subjects). The results of this study and other consistent studies show that low resilience may be a risk factor for suicidal behavior. Therefore, it seems that longitudinal studies among suicide attempters, such as depression measurement, may better evaluate the possible relationship between resilience and suicidal behavior.^26^

 Heisel and Flett defined resilience as a part of Meaning in Life. These researchers concluded that, at first, the meaning in life had a negative relationship with the start or worsening of suicidal thoughts over time, controlling risk factors and intervening accelerating factors. In addition, they showed that the meaning in life is responsible for promoting mental health and well-being and potentially creating resilience to suicidal thoughts in the future.^27^ Kleiman and Beaver conducted a study to define life as a suicidal resilience factor. They concluded that the search for meaning in life, but not its existence, mediated the relationship between interpersonal psychological theory variables and suicidal thoughts. These researchers’ findings showed that interventions targeting the meaning in life might reduce the suicide risk in people.^28^

 Resilience is also called the people’s positive capacity to adapt to stressors and disasters. People with high resilience can overcome all kinds of adversarial effects. People with low resilience may use regressive coping strategies in dealing with stressful events. These people are likely to tend to passivity and churn or withdraw behaviorally and cognitively. Resilience acts as a protective factor against stressful factors.

 In conclusion,an inverse and significant relationship was observed between resilience and suicidal thoughts in men and women. Therefore, further assessments are required to investigate the lower resilience reasons, including depression or lack of skills, and take measures to increase it among young people.
